# Direct structural identification of carbenium ions and investigation of host–guest interaction in the methanol to olefins reaction obtained by multinuclear NMR correlations†
†Electronic supplementary information (ESI) available. See DOI: 10.1039/c7sc03657d


**DOI:** 10.1039/c7sc03657d

**Published:** 2017-10-10

**Authors:** Dong Xiao, Shutao Xu, Xiuwen Han, Xinhe Bao, Zhongmin Liu, Frédéric Blanc

**Affiliations:** a State Key Laboratory of Catalysis , Dalian Institute of Chemical Physics , Chinese Academy of Sciences , 457 Zhongshan Road , Dalian 116023 , China; b University of Chinese Academy of Sciences , Beijing 100049 , China; c Department of Chemistry , University of Liverpool , Crown Street , Liverpool , L69 7ZD , UK . Email: frederic.blanc@liverpool.ac.uk; d National Engineering Laboratory for Methanol to Olefins , Dalian National Laboratory for Clean Energy , Dalian Institute of Chemical Physics Chinese Academy of Sciences , Dalian 116023 , China; e Stephenson Institute for Renewable Energy , University of Liverpool , Crown Street , Liverpool , L69 7ZD , UK

## Abstract

Probing and determining the intermediates formed during catalytic reactions in heterogeneous catalysis are strong challenges. Using ^13^C labelling and two dimensional ^13^C–^13^C through-bond NMR correlations, we directly reveal the structures of a range of carbenium ion species formed during the conversion of methanol to olefins on acidic H-ZSM-5 zeolite by mapping the carbon–carbon bond connectivities. Additionally, we use ^13^C–^27^Al and ^29^Si–^13^C through-space NMR experiments to probe the interactions between the confined carbon species (including carbenium ions) and the framework of the zeolite, which quantitatively provide an estimate for the carbon–aluminium and carbon–silicon distances, respectively.

## Abstract 2

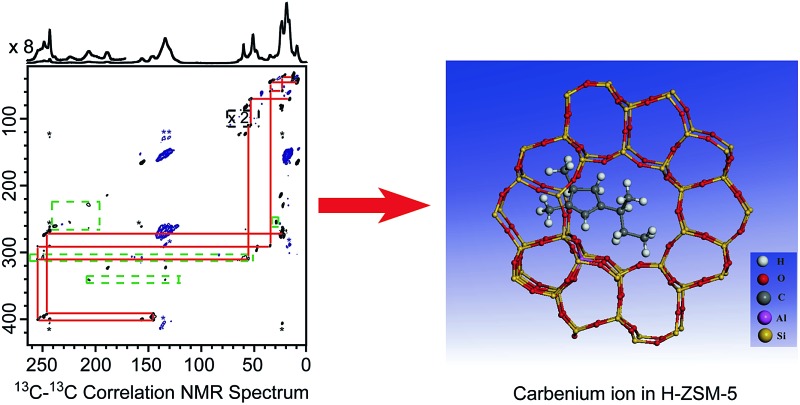
Structural identification of carbenium ion intermediates and quantitative investigation of their interactions with zeolite H-ZSM-5 by multinuclear MAS NMR.

## Introduction

The methanol-to-olefins (MTO) reaction is an important process for the production of light olefins (mainly ethylene and propene) from non-petrochemical resources such as coal and natural gas.^1^ The reaction is catalysed by microporous solid acids, in particular a wide range of zeolites (*e.g.* H-ZSM-5 and H-SAPO-34), and has been successfully commercialised since the 1990s.^1^–^3^ Nevertheless, there is a need for a deeper understanding of the catalytic active sites and reaction mechanism in order to identify catalyst deactivation pathways and further optimise the catalytic performance.^4^–^11^

Solid-state NMR is a well-developed technique for structural determination and host–guest investigation studies, and has played an important role in increasing our understanding of heterogeneous catalytic processes.^12^–^15^ In particular, solid-state NMR has enabled the detection of the intermediates formed during the MTO reaction and their interactions with zeolite, which are both important in unravelling the mechanism of the reaction. The intermediates that have been observed previously by NMR are cyclic carbenium ions such as polymethylcyclopentenyl cations and polymethylbenzenium cations on H-SAPO-34,^16^ H-SSZ-13,^16^ DNL-6 (ref. 17) and β-zeolite,^18^ and polymethylcyclopentenyl cations and ethylated cyclopentenyl cations on H-ZSM-5 zeolite.^19^,^20^ These cyclic carbenium ions are crucial intermediates involved in the hydrocarbon pool mechanism^21^ in which the cyclic organic species in the zeolite pores act as co-catalysts for the conversion of methanol to olefins.^5^ More specifically, two reaction routes have been proposed namely the side-chain methylation route in which the olefins are produced through the methylation of polymethylbenzenium ions and the subsequent elimination of the side chain groups, and the paring route in which the olefins are released *via* the expansion of polymethylcyclopentenyl cations and the subsequent contraction of polymethylbenzenium ions.^16^,^22^–^24^ The structural identification of the carbenium ions is crucial for the determination of the dominant route and specific reaction path in different zeolites.

Previous work relied on computational methods for the assignments of the ^13^C solid-state NMR spectra of the carbenium ions.^16^,^19^ Although these computational methods are recognised as robust approaches for NMR spectral interpretation,^25^,^26^ there is still a direct lack of experimental data supporting these assignments and therefore this may lead to possible misinterpretation of the carbenium ions produced. Additionally, the identification of the carbenium ions was indirectly obtained by digesting the dienes (the deprotonated counterparts of the cyclic carbenium ions) with concentrated sulfuric acid and analysing the obtained solutions by liquid-state NMR.^16^,^20^ This procedure assumes that the states of the carbenium ions in the solutions are the same as those confined in the solid zeolite pores, and also requires independent synthesis of the dienes, and therefore entails prior knowledge of the possible carbenium ions’ structures.

Recently, the interactions between the carbon species and the Al sites of the H-ZSM-5 zeolite were qualitatively investigated using spatially encoded ^13^C–^27^Al dipolar coupling NMR experiments (employing a S-RESPDOR, Symmetry-based Resonance-Echo Saturation-Pulse DOuble-Resonance sequence^27^). The work demonstrated the formation of supramolecular reaction centres composed of confined carbon species and the inorganic framework of zeolite which possesses higher reactivity toward methanol in the H-ZSM-5 zeolite.^7^

Here, we unambiguously experimentally identified several cyclic carbenium ions on MTO activated H-ZSM-5 zeolite, including a previously undetected 1,5-dimethyl-3-*sec*-butyl cyclopentenyl cation, using a refocused INADEQUATE (Incredible Natural Abundance DoublE QUAntum Transfer Experiment)^28^ NMR sequence. This experiment relies on scalar J couplings and yields through-bond correlations, providing a straightforward pathway for ^13^C spectral assignments. Moreover, the interactions between the confined carbon species and the H-ZSM-5 zeolite framework are quantitatively probed *via* through-space ^13^C{^27^Al} S-RESPDOR^27^,^29^ and ^29^Si{^13^C} REDOR (Rotational Echo DOuble Resonance) experiments (see Fig. S1 in the ESI†).^30^

## Results and discussion

### Structural identification of the confined carbon species in MTO activated H-ZSM-5

**Fig. 1:**
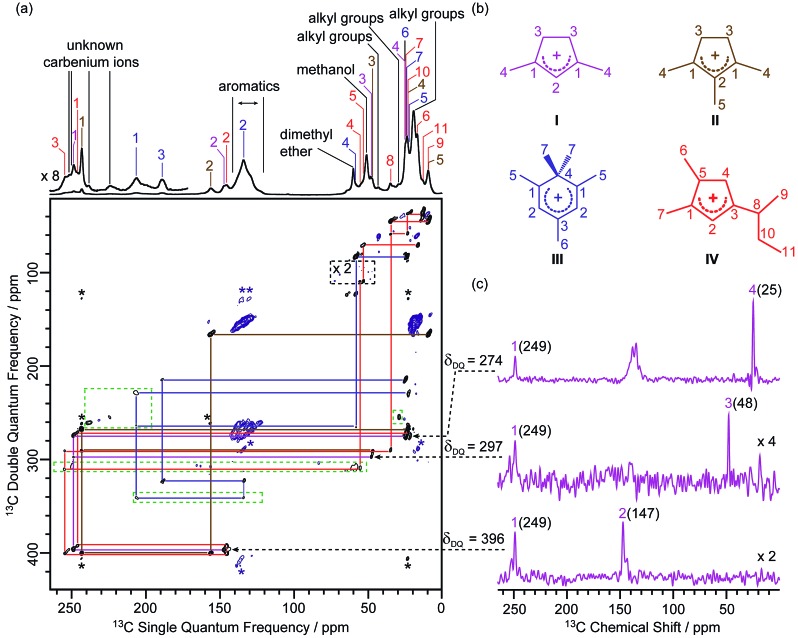
(a) 2D ^13^C–^13^C refocused INADEQUATE spectrum of ^13^C enriched MTO activated H-ZSM-5 at *B*_0_ = 9.4 T and at a MAS frequency of *v*_r_ = 14 kHz. The signals in the black dashed box have been magnified by a factor of 2, while signals in the green dashed boxes have been processed with a smaller number of *t*_1_ points and larger line broadening to account for the shorter *T*′_2_ values (see Table S4† for details) and weak intensities of these ^13^C signals to make them more easily visible. Signals corresponding to carbenium ions (black) and to other neutral carbon species (blue) are highlighted to distinguish them. The assignments of the different carbenium species are given in different colours. Asterisks (*) denote spinning sidebands. (b) Molecular structures of the carbenium ions are identified, colour-coded according to their assignments. (c) Extracted horizontal traces of carbenium ion **I** with arrows in dashed lines indicating their positions in the 2D map. The corresponding double quantum frequency *δ*_DQ_ of each slice is also given in the figure. The chemical shifts of different ^13^C sites are given in parenthesis. Unlabelled peaks are from other carbenium ions or aromatic species. Traces obtained for other identified carbeniums ions are shown in Fig. S3–S5.†

In this work, the MTO activated H-ZSM-5 materials were prepared by passing ^13^C enriched CH_3_OH over H-ZSM-5 at 285 °C for 20 minutes followed by quenching the reaction mixture with liquid N_2_ (see the Experimental section in the ESI†). The ^13^C CP (Cross-Polarisation) MAS (Magic Angle Spinning) NMR spectra of the ^13^C enriched MTO activated H-ZSM-5 are given in Fig. 1a at 9.4 T (and in Fig. S2† at 20 T) and show multiple signals ranging from 0 to 260 ppm, highlighting the complexity of the confined carbon species. It is worth pointing out that the unusual downfield ^13^C resonances (235–260 ppm) are characteristic signals of protonated carbenium ions and these have previously been assigned to several methylated and ethylated cyclopentenyl cations^19^,^20^ whose overlapping resonances prevent their unequivocal assignments.

A 2D ^13^C–^13^C refocused INADEQUATE spectrum is displayed in Fig. 1a and gives correlations mapping out the carbon skeleton of each of the carbenium ions. In this J-based experiment, two directly bonded ^13^C nuclei share a common frequency in the double quantum (vertical) dimension at the sum of their ^13^C individual frequencies in the single quantum (horizontal) dimension.^28^ The peaks observed in the INADEQUATE spectrum notably allowed us to explicitly identify the three methylated carbenium ions, namely the dimethylcyclopentenyl cation **I**, trimethylcyclopentenyl cation **II** and pentamethylbenzenium cation **III** (see Fig. 1b) which have been previously proposed but were identified based on a combination of 1D ^13^C CP NMR spectra, GC-MS (Gas Chromatography-Mass Spectrometry) and DFT (Density Functional Theory) calculations.^19^ More explicitly, the dimethylcyclopentenyl cation **I** can be identified through correlations C1(**I**) (249 ppm) – C2(**I**) (147 ppm), C1(**I**) (249 ppm) – C3(**I**) (48 ppm) and C1(**I**) (249 ppm) – C4(**I**) (25 ppm) as identified in purple in Fig. 1a (and in Fig. 1c for all horizontal traces). A similar approach is used to directly establish the carbon connectivities in cations **II** (Fig. S3†) and **III** (Fig. S4†).

A previously unprecedented observed 1,5-dimethyl-3-*sec*-butyl cyclopentenyl cation **IV**, is also identified on H-ZSM-5 as revealed by the C3(**IV**) (255 ppm) – C8(**IV**) (35 ppm), C8(**IV**) (35 ppm) – C9(**IV**) (9 ppm), C8(**IV**) (35 ppm) – C10(**IV**) (23 ppm), C10(**IV**) (23 ppm) – C11(**IV**) (13 ppm) correlations and correlations amongst C1(**IV**) to C8(**IV**) observed in the 2D ^13^C–^13^C refocused INADEQUATE spectra (red lines in Fig. 1a and S5†). A cyclopentenyl cation with a *tert*-butyl group has previously been reported to be involved as a key intermediate in the aromatic-based paring route proposed by theoretical modelling for the formation of isobutene in the MTO reaction, however, it was not experimentally observed.^24^ In contrast, some work proposed that butenes are formed through an alkene-based cycle involving the methylation/cracking of alkenes.^31^,^32^ Hence, the experimental identification of cation **IV** provides direct support for the aromatic-based paring route for butene formation in H-ZSM-5. The elimination of the *sec*-butyl group of cation **IV** is likely to produce but-1-ene and but-2-ene which are also the products of the MTO reaction.^33^

Several correlations relating to ^13^C signals in the 235 ppm to 260 ppm region (black lines in Fig. S6†) are also obtained and can be assigned to some additional carbenium ions, most likely cyclopentenyl cations.^19^,^20^ The correlation involving the weak signal at 225 ppm may arise from the polymethylcyclohexenyl cations.^34^ However, the lack of correlations relating these signals with the aliphatic region of the ^13^C NMR spectrum limits the complete assignments of these signals and is probably due to the low concentration of these carbenium ions as evidenced by their weak signal intensities in the 1D ^13^C CP MAS spectrum. The low concentration may also account for the apparent absence of ethylated cyclopentenyl cations^20^ in our MTO activated H-ZSM-5.

The increase in resolution offered in the vertical dimension of the 2D spectrum enables a more accurate determination of the ^13^C chemical shift values of the different carbon sites from these carbenium ions (Table S4†). Those signals are usually poorly resolved in the 1D CP MAS NMR spectrum (Fig. 1a and Fig. S2† for data at 9.4 T) even at a high magnetic field (see Fig. S2†).

In the 2D ^13^C–^13^C refocused INADEQUATE spectrum, signals ranging from 120 to 140 ppm show strong correlations with each other and with signals in the 13–22 ppm region (maroon lines in Fig. S6†). These correlations are attributed to correlations amongst carbons of the benzene rings and between benzene ring carbons and alkyl group carbons of neutral aromatic species, respectively. Aromatics with various types and numbers of substituted alkyl groups have very close chemical shifts,^35^ which makes individual assignments of signals from these aromatics challenging due to a lack of resolution.

Signals at 60 and 51 ppm have strong intensities in the 1D CP MAS spectrum and show no correlations with any other signals in the 2D spectrum (Fig. 1a). This observation is consistent with their assignments to dimethyl ether and residual adsorbed methanol, respectively.^20^ Correlations among peaks between 10 and 45 ppm (orange lines in Fig. S6†) can be assigned to alkyl groups of aromatics or carbenium ions.^20^

### Quantitative investigation of the interactions between the confined carbon species and the H-ZSM-5 framework

**Fig. 2:**
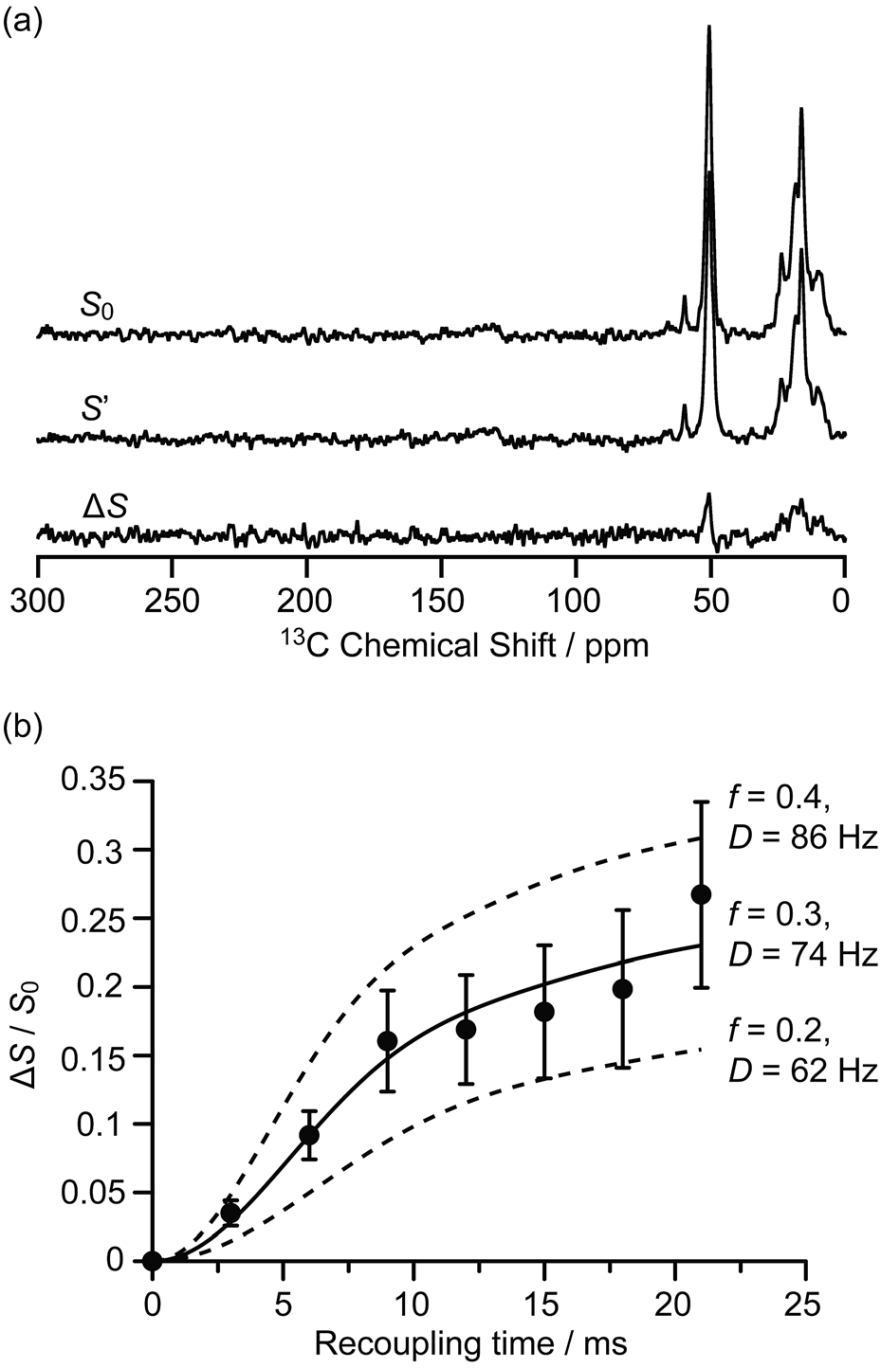
(a) ^13^C{^27^Al} S-RESPDOR signals with (*S*′) and without (*S*_0_) ^27^Al irradiation at an external magnetic field *B*_0_ = 9.4 T and with a recoupling time of 15 ms. Δ*S* is the difference spectrum between *S*_0_ and *S*′. (b) ^13^C{^27^Al} S-RESPDOR fraction Δ*S*/*S*_0_ as a function of the recoupling time with the corresponding best-fit curve (black line) and fit boundaries (dashed lines). *f* is the pre-factor and *D* is the dipolar coupling constant (see ESI†). The error bars are determined from the signal to noise ratios of the *S*_0_ and *S*′ spectra as measured by the TopSpin3.2 NMR software.

The interactions between the confined carbon species and zeolite are initially investigated by recording ^13^C{^27^Al} S-RESPDOR data, in which the ^13^C–^27^Al dipolar couplings are reintroduced by the SR421 recoupling sequences^36^ on the ^13^C spins, and provide access to the intramolecular distance. With ^27^Al irradiation and at a recoupling time of 15 ms, the S-RESPDOR dephasing Δ*S*/*S*_0_ obtained at 9.4 T can be clearly observed, indicating spatial proximities between the ^13^C and ^27^Al spins. More specifically, signals from 0 to 40 ppm, corresponding to the alkyl groups of both carbenium ions and aromatics, all have similar signal reduction Δ*S*/*S*_0_ due to being coupled to the ^27^Al spins (Fig. 2a) and are integrated against the recoupling time in Fig. 2b. Due to the small concentration of ^27^Al atoms in ZSM-5 (SiO_2_/Al_2_O_3_ = 50), the ^13^C spins are unlikely to be coupled with multiple ^27^Al spins and a single spin pair model is used to fit the S-RESPDOR data^27^ (see ESI† for further details). A ^13^C–^27^Al dipolar coupling constant *D* of 74 ± 12 Hz is extracted and corresponds to an average ^13^C–^27^Al distance of 4.7 ± 0.3 Å between the alkyl groups of the confined carbon species and the ^27^Al sites, and the ^27^Al NMR spectrum in Fig. S7† shows mainly the framework tetrahedral Al.

**Fig. 3:**
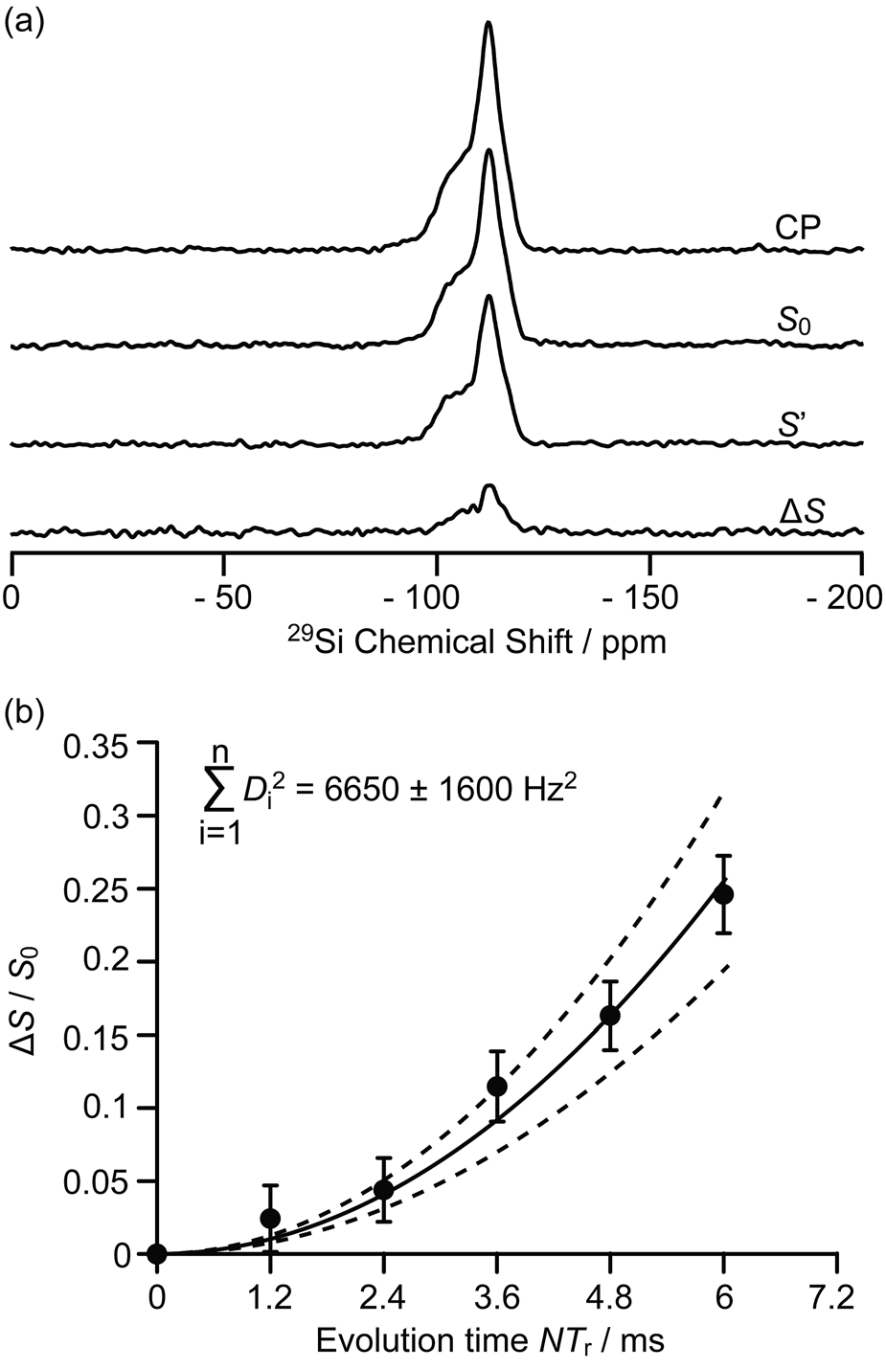
(a) ^29^Si CP, ^29^Si CP spin echo signal (*S*_0_) and ^29^Si{^13^C} REDOR signal with the reintroduction of dipolar couplings (*S*′) at an external magnetic field *B*_0_ = 9.4 T and with an evolution time *NT*_r_ of 6 ms (*T*_r_ is the rotor period, *N* is the number of rotor periods, see Fig. S1†). Δ*S* = *S*_0_ – *S*′. (b) Plot of the REDOR fraction Δ*S*/*S*_0_ as a function of the evolution time *NT*_r_ with the corresponding best-fit curve (black line) and fit boundaries (dashed lines). Vertical error bars are estimated as for the ^13^C{^27^Al} S-RESPDOR data.

The 9.4 T ^29^Si CP MAS NMR spectra of the MTO activated H-ZSM-5 (Fig. 3a and S8†) show multiple broad signals at –102, –106, –112 and –117 ppm which are characteristic of the (SiO)_3_SiOH (Q^3^), Si(OSi)_3_(OAl), Si(OSi)_4_ (Q^4^) and the crystallographically inequivalent Si(OSi)_4_ (Q^4^′) sites, respectively, of which the Si(OSi)_3_(OAl) sites contribute to the Brønsted acid sites.^37^ Note that no ^29^Si signal for the T_*n*_ sites of the type R–Si(OSi)_*n*_(OH)_3–*n*_ (typically observed around –60 ppm (ref. 38)) could be detected on H-ZSM-5, indicating that the confined carbon species are not directly covalently bonded to the ^29^Si nuclei.

Spatial interactions between the confined carbon species and the H-ZSM-5 zeolite framework are further probed by ^29^Si detected ^29^Si{^13^C} REDOR experiments (Fig. 3a) which reintroduce the ^29^Si–^13^C dipolar couplings under MAS.^30^ At an evolution time of 6 ms, the intensity of the dephased ^29^Si NMR signal *S*′ is significantly reduced *vs.* the spin echo signal *S*_0_, indicating spatial proximities between ^29^Si and ^13^C spins. Fig. S8† shows that the different ^29^Si signals have a similar degree of intensity reduction and these signals cannot be well resolved even at a high field of 20 T (Fig. S9†). Therefore, integration of the whole ^29^Si NMR signals from –90 to –125 ppm was used to determine the REDOR fraction Δ*S*/*S*_0_ as a function of the evolution time (Fig. 3b). The number of retained carbon species and their unknown geometries with respect to the zeolite impose the use of a geometrically-independent REDOR curve model in which only data for a short dipolar evolution time (Δ*S*/*S*_0_ < 0.3) are needed.^39^

Fitting the ^29^Si{^13^C} REDOR data (see Fig. 3b and ESI†) yields ∑*D*_*i*_^2^ of 6650 ± 1600 Hz^2^ which gives an estimated ^29^Si–^13^C dipolar coupling constant *D* of 82 ± 10 Hz (assuming a simplified single spin pair model) and a ^29^Si to ^13^C internuclear distance of 4.2 ± 0.2 Å. This distance is comparable to the one obtained above for ^13^C to ^27^Al from the ^13^C{^27^Al} S-RESPDOR experiments, showing strong interactions between the confined hydrocarbon species and zeolite framework and providing quantitative information for the proposed supramolecular reaction centres in H-ZSM-5.^7^,^40^

The interactions between the neutral aromatics, carbenium ions and H-ZSM-5 have previously been investigated computationally.^41^–^45^ It was found that it is the confinement of pores *via* long-range van der Waals interactions between the neutral aromatics and zeolite framework that contributes considerably more to the aromatics’ adsorption in H-ZSM-5 than the short-range interactions between the acid OH group of the zeolite and the electrons of the aromatic ring. These previous works proposed that the aromatics prefer to adsorb in the intersection region between the straight and sinusoidal channels in which polycyclic aromatics grow and block the channels, leading to the catalysts’ deactivation.^41^–^43^ The ^29^Si{^13^C} REDOR spectra (Fig. S8†) show that different ^29^Si sites, including the Si(OSi)_3_(OAl) sites corresponding to the Brønsted acid sites, have apparent similar interactions with ^13^C nuclei, which indicates that the confinement effects dominate the adsorption of the main hydrocarbon species (neutral aromatics), and that the short-range interactions between the main hydrocarbon species and the Brønsted acid sites may not be strong enough to make a significant difference between these acid sites (and others). These observations suggest that the deactivation of zeolite may not result from the direct poisoning of acid sites, but from blockage of the channels due to the accumulation of aromatics, which is consistent with previous calculations.^41^ The adsorption model in previous studies showed that the acid O–H bond axis faces the aromatic ring in a nearly perpendicular orientation with the distance between the acidic H and the aromatic ring falling in the 2.2–2.9 Å range.^41^ Considering an Al–H distance of about 2.4 Å (ref. 43) and the size of aromatics, both ^27^Al–^13^C and ^29^Si–^13^C distances around 4–5 Å can be expected, matching the values measured above.

Carbenium ions were previously proposed to form ion-pair complexes with the Brønsted acid sites. In the DFT optimised geometry of this complex, the ^13^C nucleus directly involved in the ionic bonding interaction is around 3.1 Å away from the O of the Brønsted acid sites. Considering the Al–O and Si–O bond distances (1.7 and 1.6 Å respectively)^44^,^45^ and the local geometry of the complex, distances between this ^13^C nucleus and ^27^Al/^29^Si can be estimated to be around 4 Å. This ^13^C nucleus is on the carbenium ring in the optimised geometry and should be the closest one to the Al sites. Hence, we can expect a longer distance between the dangling alkyl groups of the carbenium ions and the Al sites, satisfying the experimental value of 4.7 ± 0.3 Å distance as measured by the ^13^C{^27^Al} S-RESPDOR experiments.

## Conclusions

In conclusion, we show here that a ^13^C–^13^C refocused INADEQUATE experiment on a MTO activated H-ZSM-5 leads to the unambiguous assignment of the ^13^C NMR spectrum and the direct spectroscopic determination of the molecular structures of the retained carbon species inside the zeolite framework. The spatial proximities between these carbon species and the zeolite framework were probed by ^13^C{^27^Al} S-RESPDOR and ^29^Si{^13^C} REDOR experiments for which quantitative analysis reveals carbon–aluminium and carbon–silicon host–guest distances in the range of 4.2–4.7 Å, supporting pore confinement interactions (Fig. S10†).

## Conflicts of interest

There are no conflicts to declare.

## Supplementary Material

Supplementary information
