# Supplementary material for: Direct structural identification of carbenium ions and investigation of host–guest interaction in the methanol to olefins reaction obtained by multinuclear NMR correlations
Source: Chem Sci. 2017 Oct 10;8(12):8309–14. doi: 10.1039/c7sc03657d (PMC5858018; doi:10.1039/c7sc03657d)
Supplement: Supplementary file 1 [file SC-008-C7SC03657D-s001.pdf]

Electronic Supplementary Material (ESI) for Chemical Science.
This journal is © The Royal Society of Chemistry 2017

Supplementary Information for:

Direct structural identification of carbenium ions and investigation of host-guest interaction in the methanol to olefins reaction obtained by multinuclear NMR correlations

Dong Xiao,[1,2,3] Shutao Xu,[4] Xiuwen Han,[1] Xinhe Bao,[1] Zhongmin Liu,[1,4] and Frédéric Blanc*[3,5]

[1] State Key Laboratory of Catalysis, Dalian Institute of Chemical Physics, Chinese Academy of Sciences, 457 Zhongshan Road, Dalian 116023 (China)

[2] University of Chinese Academy of Sciences, Beijing 100049 (China)

[3] Department of Chemistry, University of Liverpool, Crown Street, Liverpool, L69 7ZD (UK)

[4] National Engineering Laboratory for Methanol to Olefins, Dalian National Laboratory for Clean Energy, Dalian Institute of Chemical Physics Chinese Academy of Sciences, Dalian 116023 (China)

[5] Stephenson Institute for Renewable Energy, University of Liverpool, Crown Street, Liverpool, L69 7ZD (UK)

* To whom correspondence should be addressed (F.B.).

E-mail: frederic.blanc@liverpool.ac.uk.

Experimental Section

Preparation of MTO (Methanol To Olefins reaction) activated samples

H-ZSM-5 (SiO_2/Al_2O_3 = 50, from Nankai University Catalyst Co., Ltd) of 40 ~ 60 mesh particles was placed at a fixed-bed quartz tubular reactor, and was dehydrated prior to reaction by heating at 550 ^oC under a continuous flow of pure helium flow for 2h. After dehydration, the temperature was gradually decreased to the reaction temperature of 285 ^oC. The ^{13}C enriched methanol (99 atom % ^{13}C, Sigma-Aldrich) was then fed by passing the carrier gas (He) through a methanol saturator maintained at 14 ^oC with the WHSV (weight hourly space velocity) of methanol to be 2.0 h^{-1}. After running at 285 ^oC for 20 minutes, the reaction was quenched by immersing the reactor into liquid N_2. At the same time, the reacting gas was switched to pure helium gas. Once back to room temperature, the reactor was transferred to the N_2 glove box, and the activated zeolite was collected and stored. Samples were vacuumized and sealed in plastic bags using a vacuum packer in the N_2 glove box for storage and transportation. Samples were packed into NMR rotors in air at room temperature.

Nuclear Magnetic Resonance data

Experimental details are given in Table S1, Table S2 and Table S3. All NMR data were processed using TopSpin3.2 NMR software.

Table S1. Acquisition parameters for ^{13}C solid-state NMR experiments

Experiment	^{13}C CP		^{13}C CP refocused INADEQUATE	^{13}C CP Hahn echo
Magnetic field / T	9.4[a]	20[b]	9.4[a]	9.4[a]
Number of scans	2560	2048	512	1280
Recycle delay / s	2.5	3	3	3
Magic angle spinning rate / kHz	14	18	14	14
^{1}H rf[c] field for 90° pulse / kHz	70	83	70	70
CP contact time / ms	2	4	2	2
^{1}H rf amplitude ramp for contact pulse / kHz	ramp70100.100[d]	ramp.100[e]	ramp70100.100[d]	ramp70100.100[d]
^{1}H rf field during contact pulse / kHz	60	70	60	60
^{13}C rf field during contact pulse / kHz	56	54	56	60
^{1}H rf field for SPINAL64[1] decoupling pulses / kHz	70	83	70	70
^{13}C rf field for 90° and 180° pulses / kHz	-	-	70	60
Rotor syncronized delays for both echos (τ_1 and τ_2) / ms	-	-	2.1(τ_1), 1.4(τ_2)	-
Δt_1 / us	-	-	14.3	-
Number of t_1 increments	-	-	442	-
Rotor syncronized echo time	-	-	-	66 μs to 9.3 ms
Number of data points	-	-		18

[a] Recorded on a Bruker Avance III HD solid state NMR spectrometer, using a 4 mm HXY probe in double resonance mode with the resonance frequencies of ^{1}H and ^{13}C to be 400.1 MHz and 100.6 MHz, respectively. [b] Recorded on a Bruker Avance III NMR spectrometer, using a 3.2 mm HXY probe in double resonance mode with the resonance frequencies of ^{1}H and ^{13}C to be 850.2 MHz and 213.8 MHz, respectively. [c] "rf" refers to radio-frequency. [d] ^{1}H contact rf field is swept from 70 to 100% of the set ^{1}H rf field linearly with 100 steps during contact pulse. [e] ^{1}H contact rf field is swept from 50 to 100% of the set ^{1}H rf field linearly with 100 steps during contact pulse.

Table S2. Acquisition parameters for ^{13}C and ^{27}Al solid-state NMR experiments

Experiment	$^{13}C\{^{27}Al\}$ CP S-RESPDOR	^{27}Al Hahn echo
Magnetic field / T	9.4[a]	20[b]
Number of scans	768 to 9216	1024
Recycle delay / s	3	0.7
Magic angle spinning rate / kHz	8	22
^{1}H rf field for 90° pulse / kHz	83	-
CP contact time / ms	2	-
^{1}H rf amplitude ramp for contact pulse / kHz	ramp70100.100	-
^{1}H rf field during contact pulse / kHz	60	-
^{13}C rf field during contact pulse / kHz	40	-
^{1}H rf field for CW[c] decoupling pulses / kHz	83	-
^{1}H rf field for SPINAL64 decoupling pulses / kHz	83	-
^{27}Al rf field for saturation pulse / kHz	40	-
^{13}C rf field for 90° and 180° pulses / kHz	31	-
^{13}C rf field for $SR4_1^2$ recoupling sequences / kHz	16	-
^{27}Al rf field for 90° and 180° pulses / kHz	-	70
Rotor syncronized echo time	-	45 μs

[a] Recorded on a Bruker Avance III HD solid state NMR spectrometer, using a 4 mm HXY probe in double resonance mode with a frequency splitter (REDOR-BOX)[2] attached to X channel, which enabled tuning and matching to both ^{13}C, ^{27}Al Larmor frequencies at 100.6 MHz and 104.3 MHz, repectively, on X channel. [b] Recorded on a Bruker Avance III NMR spectrometer, using a 3.2 mm HXY probe in single resonance mode with the resonance frequencies of ^{27}Al to be 221.6 MHz. [c] "CW" refers to continuous-wave.

Table S3. Acquisition parameters for ^{29}Si solid-state NMR experiments

Experiment	^{29}Si CP	$^{29}Si\{^{13}C\}$ CP REDOR	
Magnetic field / T	9.4[a]	9.4[a]	20[b]
Number of scans	10240	10240	2048
Recycle delay / s	3	3	3
Magic angle spinning rate / kHz	5	5	10
^{1}H rf field for 90° pulse / kHz	83	83	83
CP contact time / ms	3	3	9
^{1}H rf amplitude ramp for contact pulse / kHz	ramp.100	ramp.100	ramp.100
^{1}H rf field during contact pulse / kHz	60	60	70
^{1}H rf field for SPINAL64 decoupling pulses / kHz	83	83	83
^{29}Si rf field during contact pulse / kHz	47	47	50
^{29}Si rf field for 180° pulses / kHz	-	48	50
^{13}C rf field for 180° pulses / kHz	-	60	50
Number of data point	-	5	4
Evolution time of first data point / ms	-	1.2	1.6
Evolution time increment / ms	-	1.2	1.0

[a] Recorded on a Bruker Avance III HD solid state NMR spectrometer, using a 4 mm HXY probe in triple resonance mode with the resonance frequency of ^{1}H to be 400.1 MHz, X channel tuned to ^{13}C at 100.6 MHz, Y channel tuned to ^{29}Si at 79.5 MHz, respectively. [b] Recorded on a Bruker Avance III NMR spectrometer, using a 3.2 mm HXY probe in triple resonance mode with the resonance frequency of ^{1}H to be 850.2 MHz, X channel tuned to ^{13}C at 213.8 MHz, Y channel tuned to ^{29}Si at 168.9 MHz, respectively.

$^{13}C\{^{27}Al\}$ S-RESPDOR and $^{29}Si\{^{13}C\}$ REDOR data processing.

1. Analytical formulas for $^{13}C\{^{27}Al\}$ S-RESPDOR

The S-RESPDOR dephasing curve for a spin-1/2 nucleus coupled to any spin-*I* nucleus follows the general formula below:[2,3]

$$\frac{\Delta S}{S_0} = \frac{f}{2I + 1}\left\{2I - \frac{\pi\sqrt{2}}{4(2I + 1)}\sum_{k = 1}^{2I}[4I - 2(k - 1)]J_{1/4}\left(\frac{k\pi D\tau}{4}\right)J_{-1/4}\left(\frac{k\pi D\tau}{4}\right)\right\}$$

where I is the spin quantum number, I = 5/2 for ^{27}Al nucleus. f is the pre-factor that represents the fraction of crystallites in which the spin states of the quadrupolar nuclei have been saturated by the saturation pulses. This pre-factor is equal to 1 only for 100% abundant nucleus I compeletely saturated by the saturation pulses. D represents the dipolar coupling constant (unit in "Hz") of ^{13}C-^{27}Al spin pair, and τ is the total duration of $SR4_1^2$ recoupling sequences. $J_{\pm 1/4}$ denotes the ±1/4-order Bessel function of the first kind.

2. Analytical formulas for $^{29}Si\{^{13}C\}$ REDOR

For short dipolar evolution times ($\Delta S/S_0 < 0.3$), the REDOR curve is geometry-independent, and a first-order approximation can be applied, which gives the following formula:[4]

$$\frac{\Delta S}{S_0} = \frac{16}{15}(NT_r)^2\sum_{i = 1}^{n} D_i^2$$

where T_r, N, and D_i are the rotor period, the number of rotor periods and the dipolar coupling constants (unit in "Hz") of different ^{29}Si-^{13}C spin pairs, respectively.

Both the $^{13}C\{^{27}Al\}$ S-RESPDOR and $^{29}Si\{^{13}C\}$ REDOR data were fitted using MatLab R2016a software.

3. Relationship between the dipolar coupling constant and internuclear distance.

$$D_{IS} = -\frac{\mu_0}{8\pi^2}\frac{\hbar\gamma_I\gamma_S}{r_{IS}^3}$$

where D_{IS} and r_{IS} are the dipolar coupling constant (unit in "Hz") and internuclear distance between I and S spins, γ_I and γ_S correspond to the gyromagnetic ratios of I and S spins,

respectively, $\hbar$ is Planck's constant, and μ_0 is the vacuum permeability.

4. Determination of errors

The errors of S' and S_0 are determined from the signal to noise ratios S/N of S' and S_0 spectra as measured by the TopSpin3.2 NMR software. The noise intensities which are calculated from the intensities of signals and S/N values, are taken as the absolute errors of the signals. The values are $S'/(S/N)_{S'}$) for S' and $S_0/(S/N)_{S_0}$ for S_0. Since $\Delta S = S_0 - S'$, the absolute errors of ΔS will be $\{S'/(S/N)_{S'} + S_0/(S/N)_{S_0}\}$, which give the absolute errors of $\Delta S/S_0$ that is $\{(S'/S_0)/(S/N)_{S'} + 1/(S/N)_{S_0}\}$ and are shown as error bars in Figure 2b and Figure 3b.

The $\Delta S/S_0$ data set including the errors were fitted with MatLab and gave a dipolar coupling constant D with associated error at 95 % confidence bounds. The largest and smallest D values are taken as the boundaries, as shown as dashed curves in Figure 2b and 3b while the solid curves correspond to the best-fit of $\Delta S/S_0$ data set without considering the errors.

Table S4. ^{13}C Chemical shift of carbenium ions.

Carbenium ions	Carbon number	Chemical shift / ppm	T_2' / ms[a]
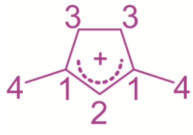 **I**	1	249	-
	2	147	-
	3	48	-
	4	25	-
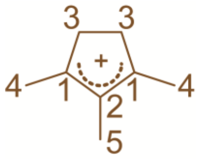 **II**	1	243	1.6
	2	156	1.6
	3	47	1.9
	4	23	2.9
	5	9	2.8
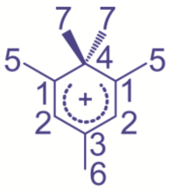 **III**	1	206	1.6
	2	134	1.2
	3	189	1.9
	4	58	-
	5	23	2.9
	6	25	-
	7	24	-
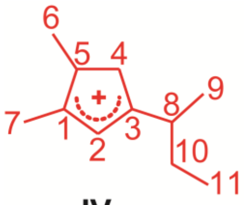 **IV**	1	246	-
	2	146	1.7
	3	255	-
	4	55	-
	5	53	-
	6	16	-
	7	24	-
	8	35	-
	9	9	2.8
	10	23	2.9
	11	13	-

[a] Measured ^{13}C transverse relaxation time (T_2') under ^{1}H decoupling (Table S1) obtained at 9.4 T. T_2' were derived by fitting the data to $I(\tau) = I(0)\exp[-\tau/T_2']$ where $I(\tau)$ and $I(0)$ are the signal intensities at time τ and $\tau = 0$.

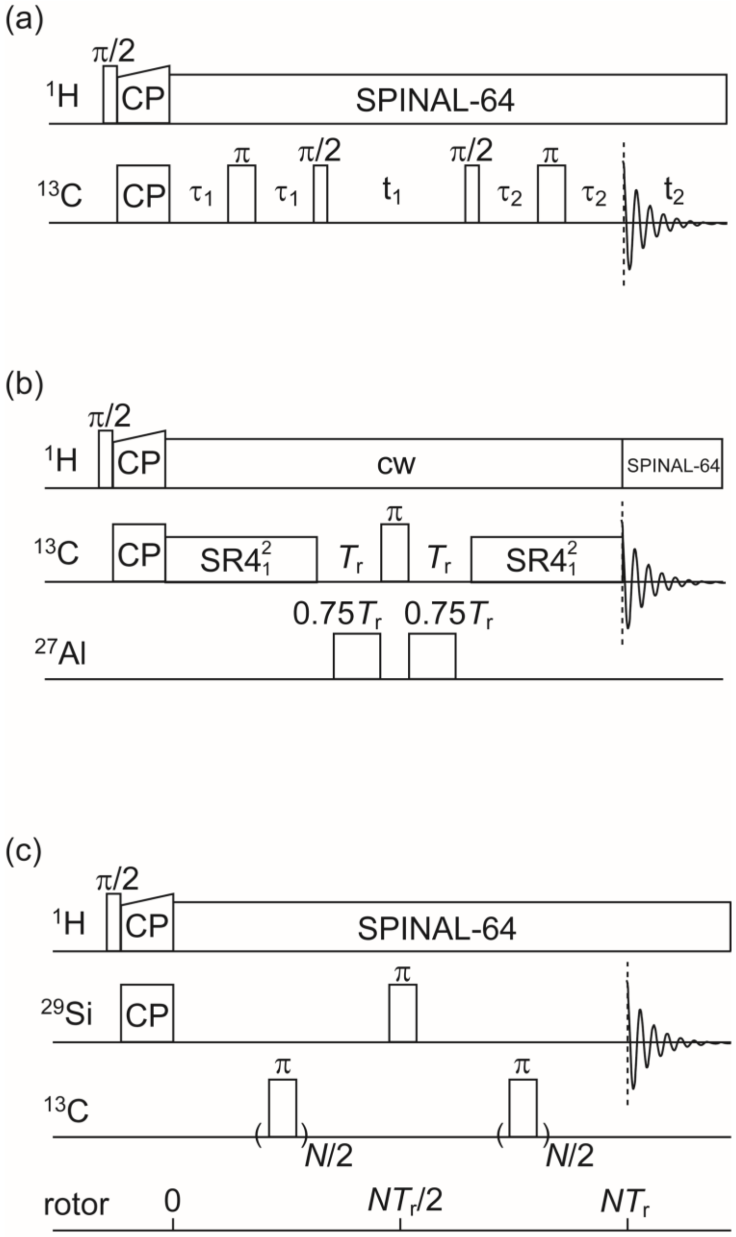

Figure S1. (a) ^{13}C CP refocused INADEQUATE pulse sequence.[5] τ_1 and τ_2 are synchronized to be an integer number of rotor periods. (b) ^{13}C{^{27}Al} CP S-RESPDOR pulse sequence.[2,3] $SR4_1^2$ is the recoupling sequence.[6] $T_r = 1/\nu_r$ is the rotor period. (c) ^{29}Si{^{13}C} CP REDOR pulse sequence. *N* is number of rotor periods. [7,8]

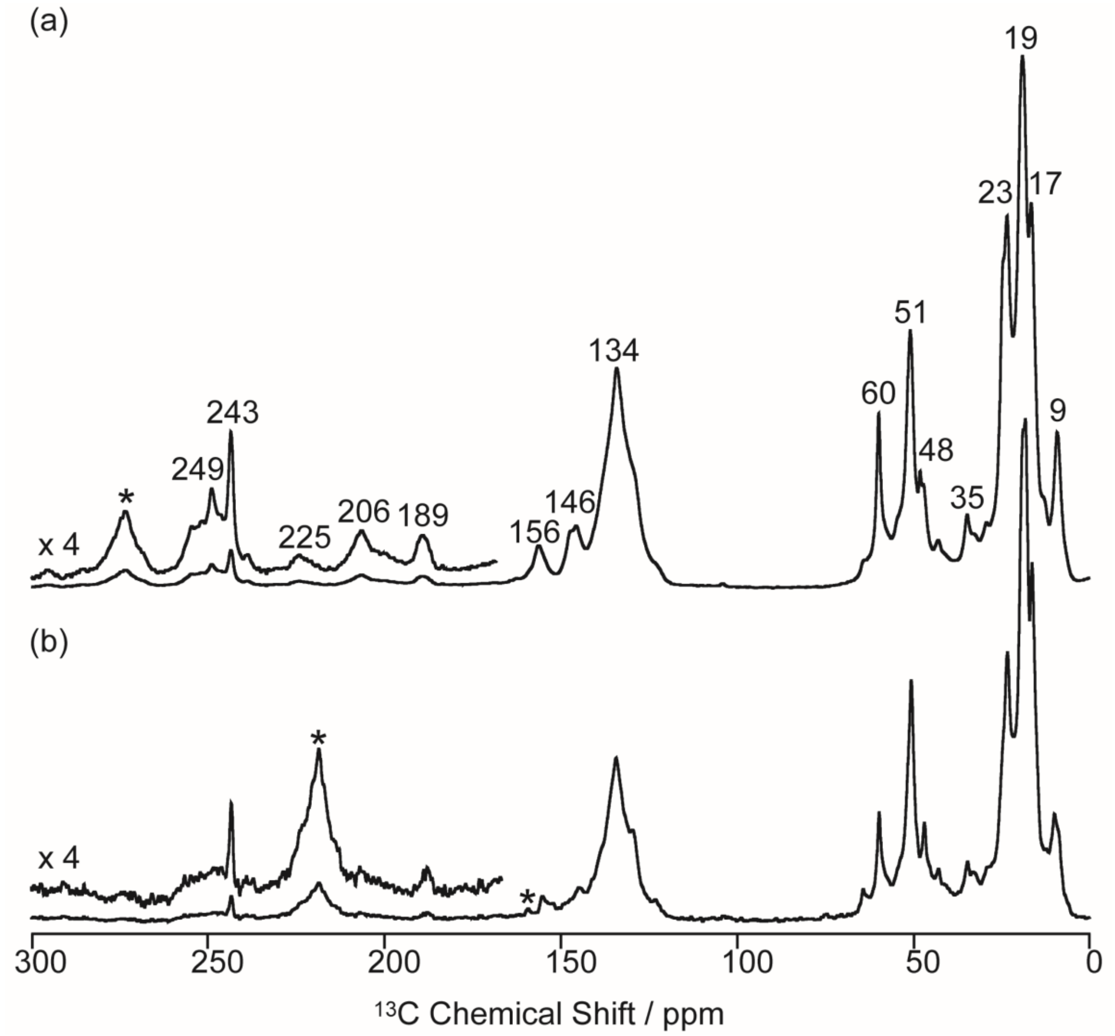

Figure S2. ^{13}C CP MAS NMR spectra of ^{13}C enriched MTO activated H-ZSM-5 ontained at (a) an external magnetic field B_0 = 9.4 T, MAS = 14 kHz and (b) B_0 = 20 T, MAS = 18 kHz. Asterisks (*) denote spinning sidebands.

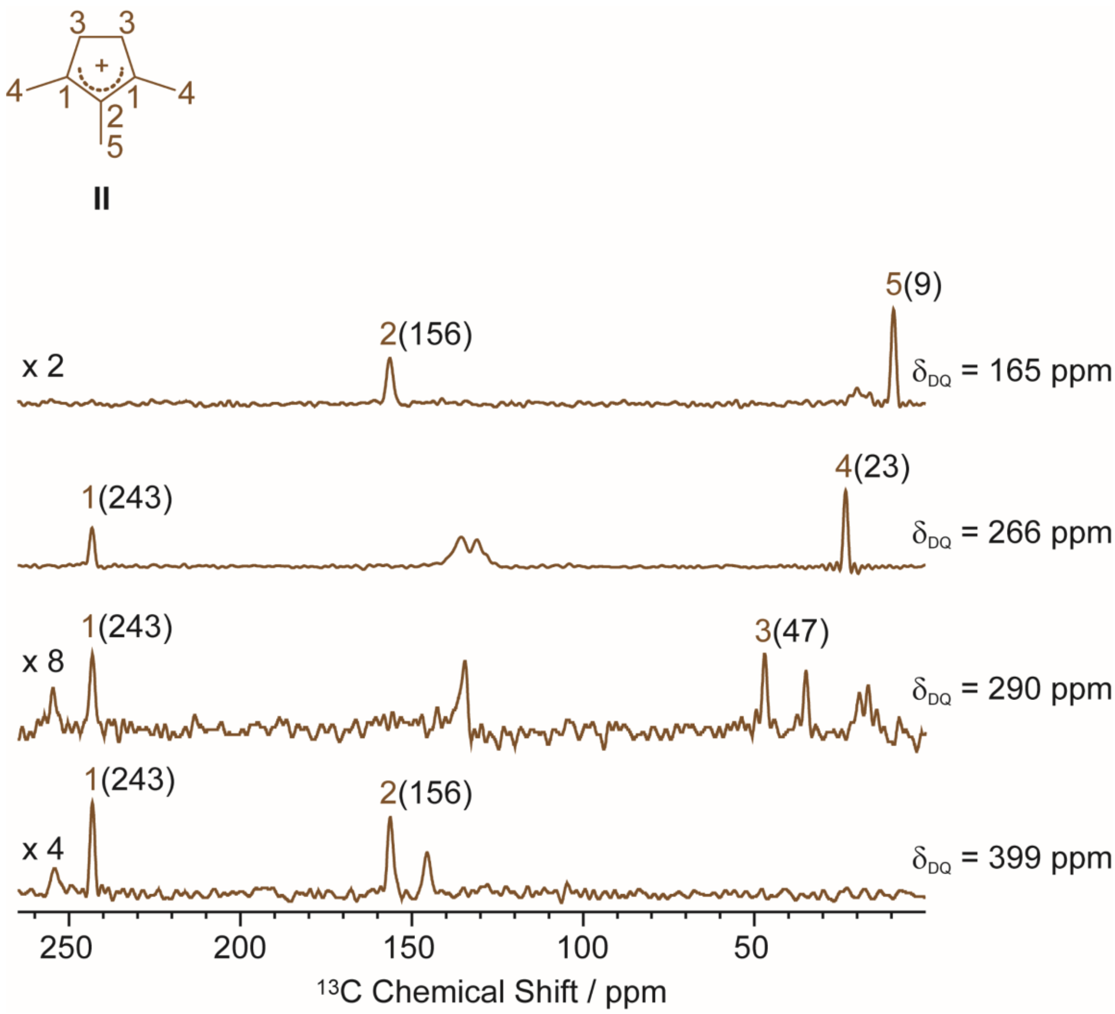

Figure S3. Extracted horizontal slices of the 2D ^{13}C INADEQUATE spectrum (Figure 1a) for carbenium ion **II**. The corresponding double quantum frequency δ_{DQ} of each slice is given in the figure. The chemical shifts of different ^{13}C sites are given in the parenthesis. Unlabelled peaks are from other carbenium ions or aromatic species.

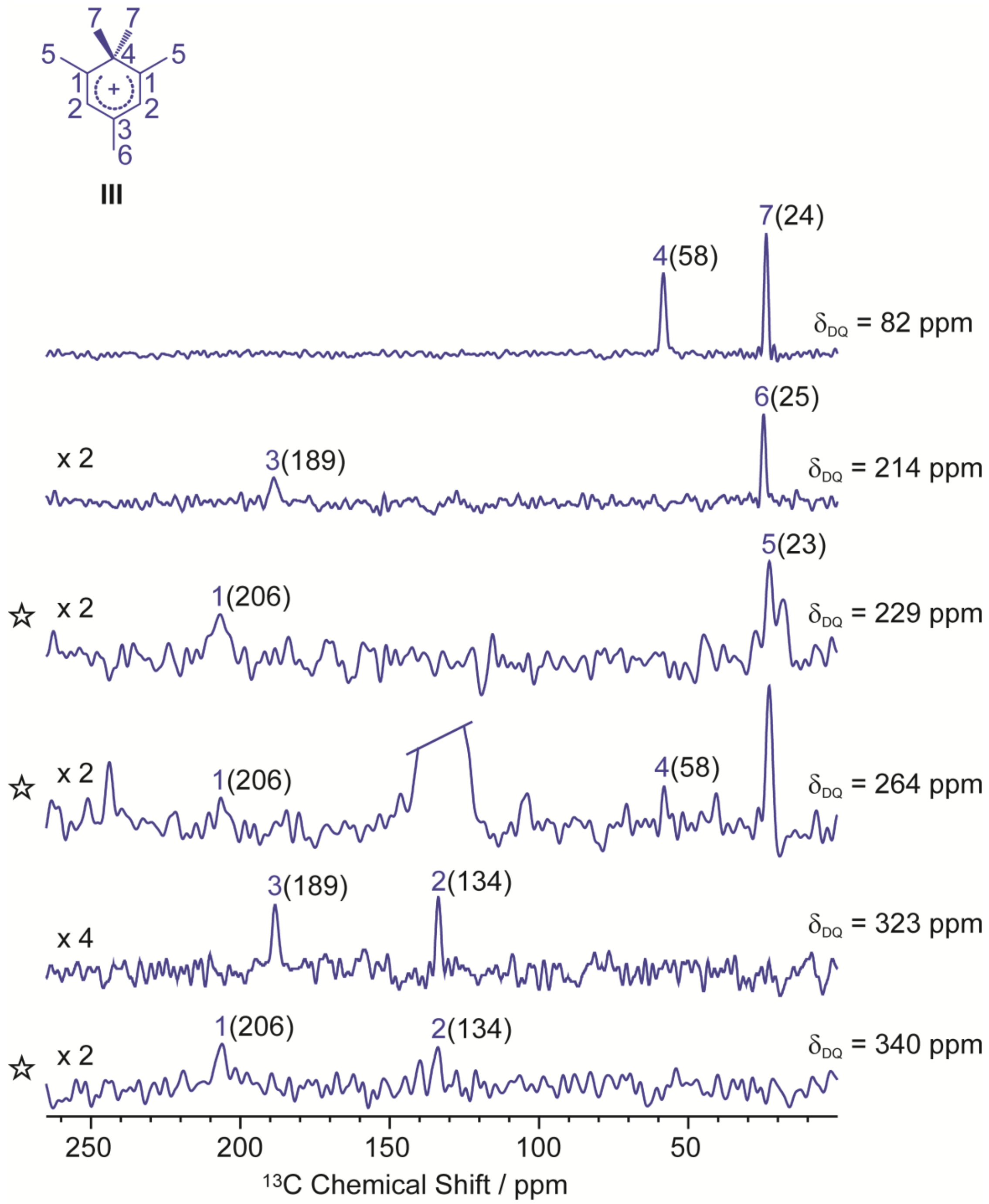

Figure S4. Extracted horizontal slices of the 2D ^{13}C INADEQUATE spectrum (Figure 1a) for carbenium ion **III**. The corresponding double quantum frequency δ_{DQ} of each slice is given in the figure. The chemical shifts of different ^{13}C sites are given in the parenthesis. Slices with "☆" on the left have been processed with a fewer number of t_1 points and larger line broadening. Unlabelled peaks are from other carbenium ions or aromatic species.

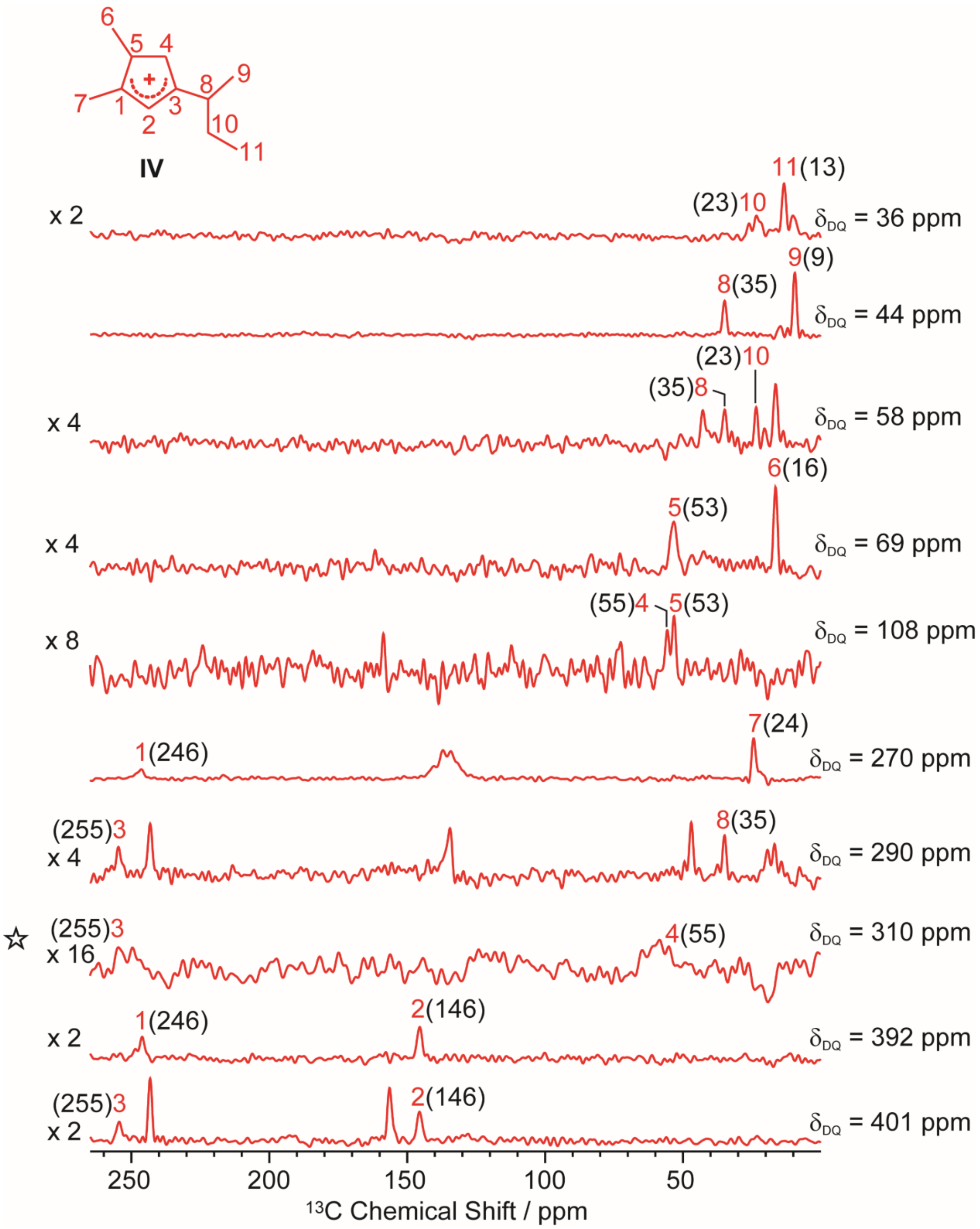

Figure S5. Extracted horizontal slices of the 2D ^{13}C INADEQUATE spectrum (Figure 1a) for carbenium ion **IV**. The corresponding double quantum frequency δ_{DQ} of each slice is given in the figure. The chemical shifts of different ^{13}C sites are given in the parenthesis. Slice with "☆" on the left has been processed with a fewer number of t_1 points and larger line broadening. Unlabelled peaks are from other carbenium ions or aromatic species.

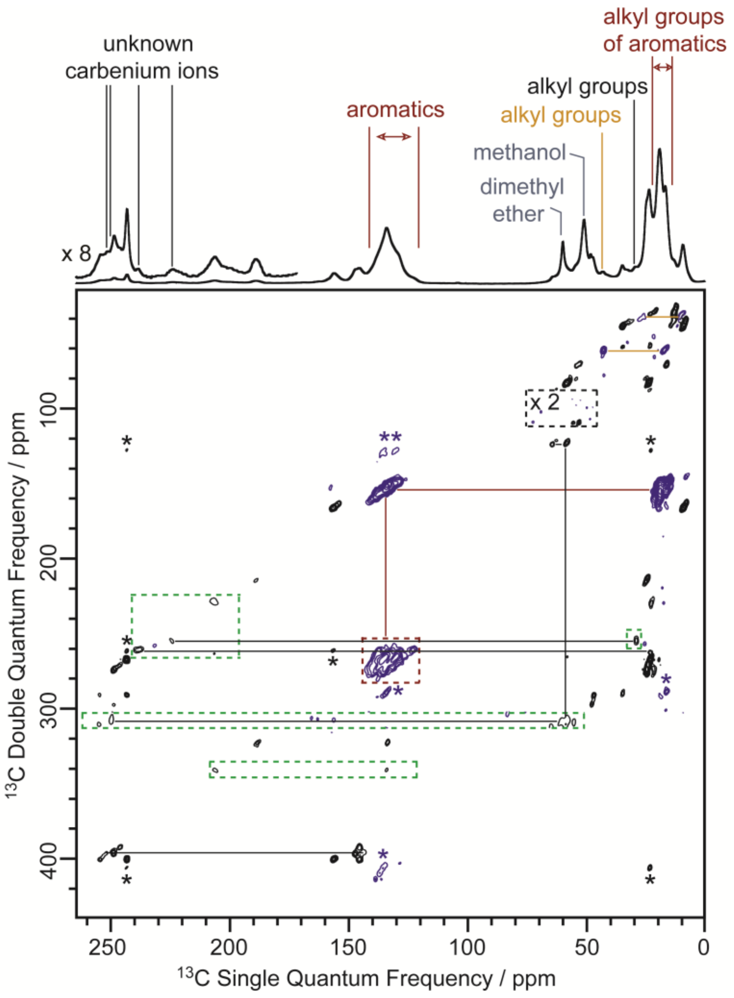

Figure S6. 2D ^{13}C-^{13}C refocused INADEQUATE spectrum of ^{13}C enriched MTO activated H-ZSM-5 at B_0 = 9.4 T and a MAS frequency of ν_r = 14 kHz. Signals in the black dashed box have been magnified by factor of 2, while signals of the green dashed boxes have been processed with a fewer number of t_1 points and larger line broadening to account for the shorter T_2' values (see Table S4 for details) and weak intensities of these ^{13}C signals and make them more easily visible. Signals corresponding to carbenium ions were labelled in black to distinguish them from signals (blue) of other neutral carbon species. Signals in maroon dashed box correspond to correlations amongst carbons of the benzene rings.[9] Asterisks (*) denote spinning sidebands.

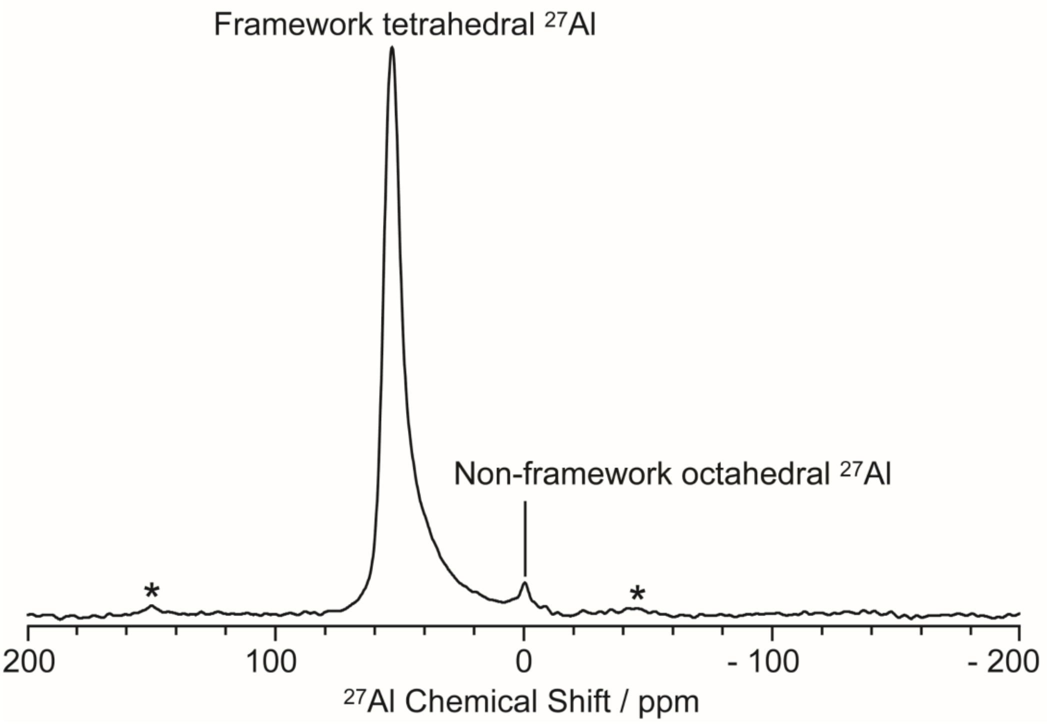

Figure S7. ^{27}Al Hahn echo MAS NMR spectrum of ^{13}C enriched MTO activated H-ZSM-5 at B_0 = 20 T. Signal are assigned according to reference[10]. Asterisks (*) denote spinning sidebands.

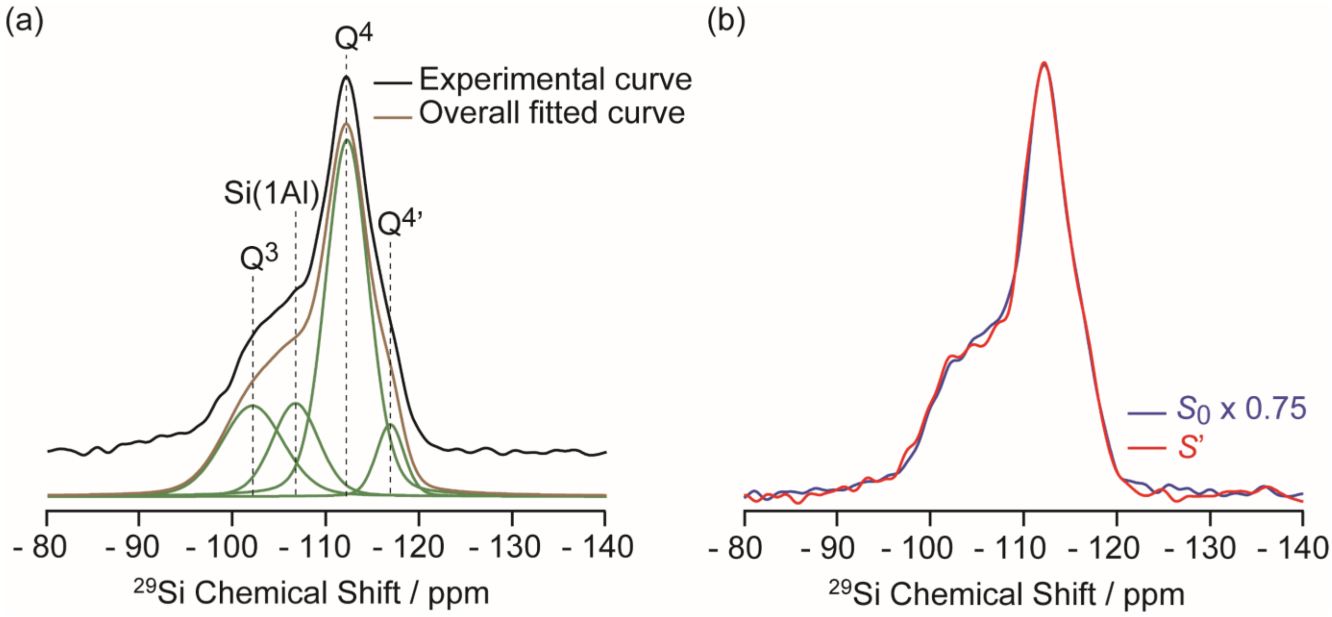

Figure S8. (a) Fitting of ^{29}Si CP spectrum at 9.4 T. Q^3, Si(1Al), Q^4 and $Q^{4'}$ correspond to $Si(OSi)_3(OH)$, $Si(OSi)_3(OAl)$ and two crystallographically inequivalent $Si(OSi)_4$ structures.[11] (b) $^{29}Si\{^{13}C\}$ REDOR signals with reintroduction of dipolar couplings (S') and without reintroduction of dipolar couplings (S_0) at external magnetic field B_0 = 9.4 T and an evolution time of 6 ms.

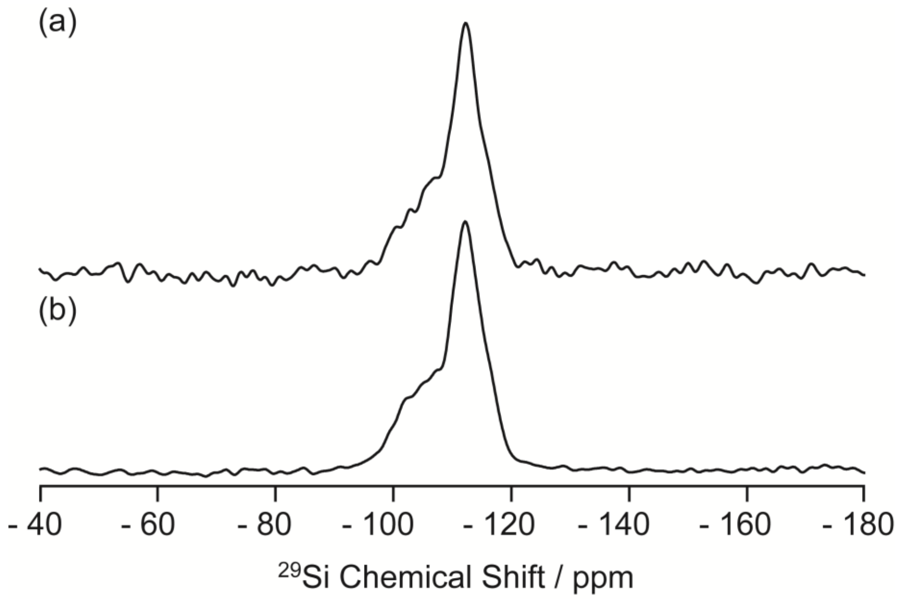

Figure S9. Comparison of $^{29}Si\{^{13}C\}$ REDOR signals (a) S_0 at 20 T, MAS = 10 kHz, NT_r = 1.6 ms and (b) S_0 at 9.4 T, MAS = 5 kHz, NT_r = 1.2 ms.

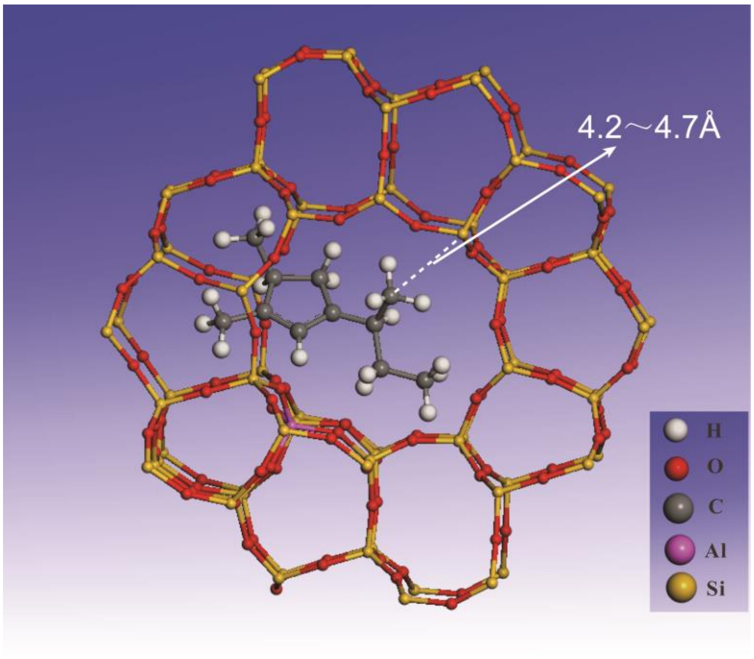

Figure S10. Representative of carbenium ion **IV** confined in the pores of H-ZSM-5 and its distance to the zeolite framework.

References

1 B. M. Fung, A. K. Khitrin and K. Ermolaev, *J. Magn. Reson.*, 2000, **142**, 97–101.

2 F. Pourpoint, J. Trébosc, R. M. Gauvin, Q. Wang, O. Lafon, F. Deng and J.-P. Amoureux, *ChemPhysChem*, 2012, **13**, 3605–3615.

3 X. Lu, O. Lafon, J. Trébosc and J.-P. Amoureux, *J. Magn. Reson.*, 2012, **215**, 34–49.

4 M. Bertmer and H. Eckert, *Solid State Nucl. Magn. Reson.*, 1999, **15**, 139–152.

5 A. Lesage, M. Bardet and L. Emsley, *J. Am. Chem. Soc.*, 1999, **121**, 10987–10993.

6 A. Brinkmann and A. P. M. Kentgens, *J. Am. Chem. Soc.*, 2006, **128**, 14758–14759.

7 T. Gullion and J. Schaefer, *J. Magn. Reson.*, 1989, **81**, 196–200.

8 T. Gullion, *Concepts Magn. Reson.*, 1998, **10**, 277–289.

9 C. Wang, Y. Chu, A. Zheng, J. Xu, Q. Wang, P. Gao, G. Qi, Y. Gong and F. Deng, *Chem. - Eur. J.*, 2014, **20**, 12432–12443.

10 D. Freude, H. Ernst and I. Wolf, *Solid State Nucl. Magn. Reson.*, 1994, **3**, 271–286.

11 Q. Wang, S. Xu, J. Chen, Y. Wei, J. Li, D. Fan, Z. Yu, Y. Qi, Y. He, S. Xu, C. Yuan, Y. Zhou, J. Wang, M. Zhang, B. Su and Z. Liu, *RSC Adv.*, 2014, **4**, 21479–21491.
